# Supplementing Freezing Medium with Crocin Exerts a Protective Effect on Bovine Spermatozoa Through the Modulation of a Heat Shock-Mediated Apoptotic Pathway

**DOI:** 10.3390/molecules30061329

**Published:** 2025-03-16

**Authors:** Vasiliki Sapanidou, Maria P. Tsantarliotou, Konstantinos Feidantsis, Eleni E. Tzekaki, Georgios Kourousekos, Sophia N. Lavrentiadou

**Affiliations:** 1 Laboratory of Animal Physiology, School of Veterinary Medicine, Faculty of Health Sciences, Aristotle University of Thessaloniki, 54124 Thessaloniki, Greece; vsapanid@vet.auth.gr (V.S.); mtsant@vet.auth.gr (M.P.T.); 2 Department of Fisheries & Aquaculture, School of Agricultural Sciences, University of Patras, 26504 Mesolonghi, Greece; kfeidant@upatras.gr; 3 Laboratory of Neurodegenerative Diseases (LND), Center for Interdisciplinary Research and Innovation, 57001 Thermi, Greece; etzekaki@chem.auth.gr; 4 Department of Reproduction and Artificial Insemination, Directorate of Veterinary Centre of Thessaloniki, National Ministry of Rural Development and Food, 57008 Thessaloniki, Greece; kourousekos@gmail.com

**Keywords:** crocin, glutathione, antioxidant capacity, iNOS, SOD-1, apoptosis, heat shock response, Hsp

## Abstract

The supplementation of freezing medium with crocin results in an amelioration of post-thawing sperm quality, as determined by motility and viability. This study aimed to examine the molecular mechanisms underlying the ameliorative effect of crocin. Bovine spermatozoa were cryopreserved in a freezing medium supplemented with 0, 0.5, or 1 mM of crocin. Sperm lysates were evaluated for their redox status and the expression of proteins implicated in the heat stress response (HSR) and apoptosis. Crocin protected spermatozoa from the accumulation of superoxide anion and ameliorated their post-thawing antioxidant capacity in terms of ROS scavenging activity and glutathione content. Moreover, crocin decreased the levels of inducible nitric oxide synthase (iNOS), while it increased superoxide dimsutase-1 (SOD-1) levels. These effects were associated with an inhibition of apoptosis, as evidenced by a decreased Bax/Bcl-2 protein ratio and decreased levels of caspase-cleaved substrates. Finally, crocin affected the heat shock response of spermatozoa, since it upregulated the levels of heat shock proteins (Hsp) 60, 70, and 90. In conclusion, the addition of crocin to the freezing medium ensured controlled amounts of ROS, enhanced the antioxidant capacity of spermatozoa, and upregulated the anti-apoptotic proteins and Hsps, thus contributing to the maintenance of cellular homeostasis.

## 1. Introduction

Fertility is inextricably associated with the maintenance of livestock populations, genetic improvement, and animal welfare. As an economically important trait, fertility is also important due to the urgent need for a more efficient, sustainable, and profitable production of food of animal origin [1]. High fertility rates and successful pregnancy outcomes are ensured with the use of frozen semen in Artificial Insemination (AI) protocols [2].

Despite the importance of cryopreservation, which is applied ubiquitously in animal and human reproduction and stem cell research, the drawbacks of this approach are still of major concern. A decline in sperm quality results in up to a 50% decrease in motility and viability [3,4]. Currently, three different mechanisms (oxidative, thermal, and osmotic stress) have been proposed to describe how cryopreservation affects the functional and structural integrity of spermatozoa [2,4]. For instance, thawing leads to the overproduction of reactive oxygen species (ROS), resulting in oxidative imbalance [5]. Although controlled amounts of ROS are required in processes crucial for the fertilizing capacity of spermatozoa, such as capacitation and acrosome reaction [6], ROS accumulation that exceeds the antioxidant capacity of spermatozoa compromises their fertilizing capacity and may lead to apoptosis [7].

ROS generation constitutes one of the main aberrations induced by the freeze/thaw process and affects every structural component of the cell. In brief, ROS accumulation and ongoing lipid peroxidation (LPO) throughout the sperm plasma membrane result in an accumulation of lipid peroxides on the sperm surface, loss of sperm motility, oxidative damage to DNA, and apoptosis [8]. Proteins, lipids, and sterols are also irreversibly affected by extreme temperature changes, which lead to ice crystal formation, osmotic stress, and changes in membrane fluidity, eventually resulting in a loss of the structural and functional integrity of the plasma membrane [2,3,4]. Unfortunately, spermatozoa lack significant antioxidant protection to counteract the damaging effect of ROS, since they discard most of their cytoplasm-containing antioxidants during the terminal stages of differentiation [9]. It has been reported by Bilodeau and co-authors that frozen/thawed bovine spermatozoa contain low levels of glutathione peroxidase (GPx), while catalase (CAT) could not be detected [10] and superoxide dismutase (SOD) is present, providing an efficient defense against ROS [11].

Cells, spermatozoa included, respond to various forms of stress by triggering or enhancing the production of a protein family known as heat shock proteins (Hsps) [12,13]. Even though the precise role of Hsps as molecular chaperones is not yet known, it has been suggested that they protect cells from the harmful effects of stressful stimuli [13,14,15], maintain cellular homeostasis, and play a crucial role in regulating cell apoptosis [16,17]. Elevated ROS concentrations and the ongoing oxidative damage to intracellular enzymes are accompanied by a decrease in Hsps [17]. Sperm cells can express Hsps pre- or post-ejaculation, indicating their role in regulating diverse sperm functions, while the role of Hsp60 in sperm capacitation and sperm–zona pellucida penetration is pivotal [18].

Several approaches have been employed to minimize ice crystal formation and oxidative injury during the freeze/thaw process, including the optimization of cryoprotectants [4,19]. However, supplementation of the extender with antioxidants seems to be the most attractive and promising strategy. Until now, scientists have not been able to agree on a specific antioxidant to be used during sperm cryopreservation or assisted reproductive techniques (ARTs) in bovines or other species. Crocin, a key bioactive water-soluble antioxidant component of saffron (*Crocus sativus* L.), and its metabolite crocetin have attracted researchers’ interest due to their several pharmacological actions [20,21,22,23]. The effects of crocin on fertility and reproductive physiology date to antiquity, since it has been used in traditional medicine to treat subfertility and as a potential sexual stimulant [24]. Recent data indicate that crocin mediates its positive effect on sperm physiology by reducing oxidative stress, which eventually improves sperm quality parameters [25,26,27]. Moreover, the post-thawing incubation of spermatozoa with crocin increases the fertilization rate in bovine species [26]. Crocin supplementation in the freezing medium of human, goat, ram, buffalo, and rooster spermatozoa was accompanied by remarkable results [19,27,28,29,30,31,32]. Crocin’s potent radical scavenger activity, particularly against superoxide anion [26,33], may underpin its ameliorative effect on sperm physiology. In addition, crocin may indirectly exert its antioxidant role through the enhancement of the activity of antioxidant enzymes and thiol content [23].

Currently, the term “freezability” has been introduced and implies the ability of spermatozoa to withstand the (thermal, osmotic, and oxidative) stress caused by cryopreservation and thawing without losing their viability and fertilizing capacity [34]. The ideal and accurate approach for assessing sperm freezability in bovine species has not been established yet, but until now, different protein or molecular biomarkers have been used to evaluate semen quality after thawing [34,35]. The same biomarkers could also be recruited for the evaluation of the efficacy of additives in the freezing extenders. In this context, and following the results of our previous research in bovine species [27], the aim of this study was to identify the molecular mechanisms adversely modulated by different concentrations of crocin and through which its supplementation in the freezing extender exerts a protective effect on bovine spermatozoa. The antioxidant properties of crocin, along with the induction of OS upon cryopreservation of spermatozoa, led us to the hypothesis that crocin may reinforce the antioxidant status of the cells. Moreover, cryopreservation leads to stress induced by extreme low temperatures, and thus, we hypothesized that heat shock proteins may be implicated in the induction of apoptosis. Therefore, we investigated the effect of crocin on the heat shock response (HSR) pathways in cryopreserved spermatozoa. To the best of our knowledge, this is the first study to investigate the potential effect of crocin on molecular pathways, such as those implicated in the heat shock response (HSR), the antioxidant enzymatic defenses of spermatozoa, such as the inducible nitric oxide synthase (iNOS) and SOD, and the apoptotic process.

## 2. Results

### 2.1. Crocin Augmented the Antioxidant Capacity and Superoxide Anion Scavenging Capacity of Bovine Spermatozoa

**Figure 1:**
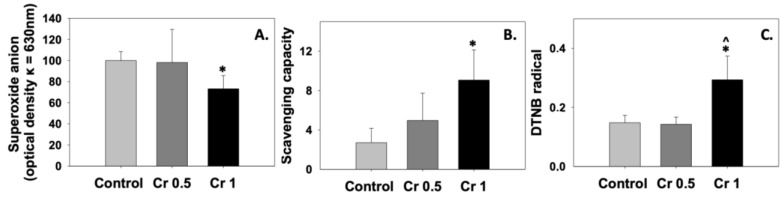
Superoxide anion (**A**), total antioxidant capacity (**B**), and intracellular GSH (**C**) levels in bovine spermatozoa under the effect of 0.5 mM (Cr 0.5) or 1 mM (Cr 1) crocin. Values constitute mean values ± S.D. of *n* = 6. The asterisk (*) denotes statistically significant differences compared to the control, while the caret (^) denotes statistically significant differences between the crocin groups (*p* < 0.05).

Figure 1 presents the antioxidant status of spermatozoa cryopreserved in the presence of crocin (Cr 0.5 and Cr 1 groups) compared to controls, as determined by the superoxide ion levels (Figure 1A), the total antioxidant capacity (TAC) of cells (Figure 1B), and intracellular GSH levels (Figure 1C). The enhancement of the TAC in the spermatozoa of the Cr 1 group (Figure 1B) was accompanied by a statistically significant (*p* < 0.05) decrease in superoxide anion production compared to the control (Figure 1A). On the other hand, 0.5 mM crocin did not affect the superoxide anion levels (Figure 1A), although the antioxidant capacity of spermatozoa increased by 45.5%, compared to the control group. Similarly, a statistically significant (*p* < 0.05) increase in the intracellular GSH levels was recorded in the presence of 1 mM crocin, compared to the control and Cr 0.5 groups (Figure 1C).

### 2.2. Crocin Retained Low Levels of iNOS and Ensured High Levels of SOD-1 in Spermatozoa

**Figure 2:**
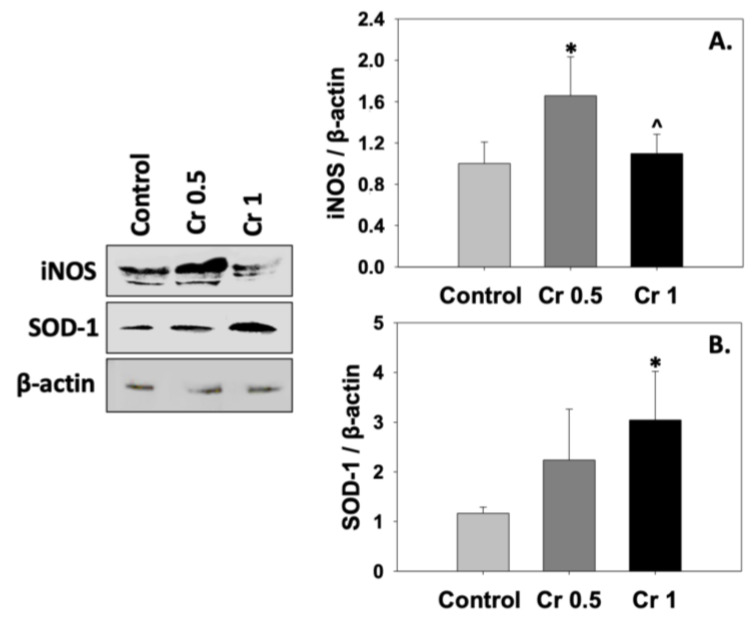
iNOS (**A**) and SOD-1 (**B**) levels in bovine spermatozoa under the effect of 0.5 mM (Cr 0.5) or 1 mM (Cr 1) crocin. Values constitute means ± S.D. of *n* = 6. Spermatozoa extracts from the control, Cr 0.5, and Cr 1 groups were immunoblotted for iNOS, SOD-1, and β-actin. Representative immunoblots are shown. The asterisk (*) denotes statistically significant differences compared to the control, while the caret (^) denotes statistically significant differences between the crocin groups (*p* < 0.05).

iNOS and SOD-1 levels are depicted in Figure 2. Spermatozoa cryopreserved in the presence of 1 mM crocin showed decreased levels of iNOS compared to the control, while statistically significant (*p* < 0.05) differences were also detected among the treated groups (Figure 2A). Moreover, SOD-1 levels were significantly (*p* < 0.05) higher in spermatozoa of the Cr 1 group compared to those of the control (Figure 2B).

### 2.3. Crocin Supplementation Enhanced HSR

**Figure 3:**
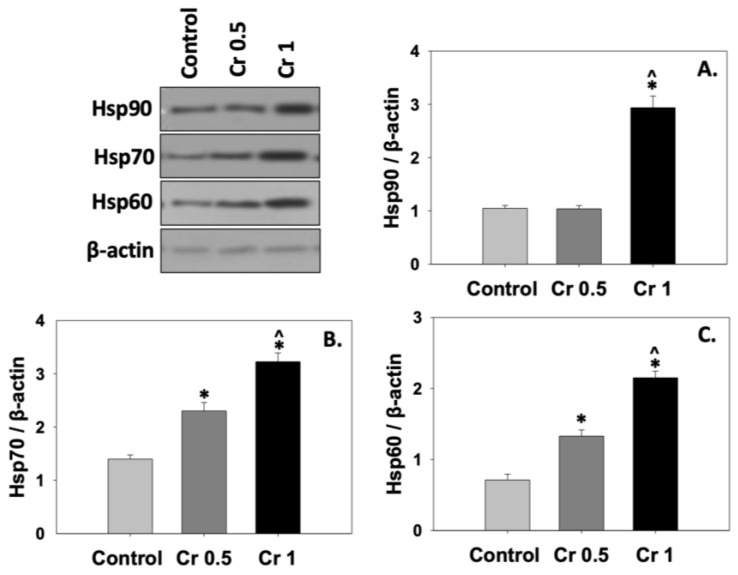
Hsp90 (**A**), Hsp70 (**B**), and Hsp60 (**C**) levels in bovine spermatozoa under the effect of 0.5 mM (Cr 0.5) or 1 mM (Cr 1) crocin. Values constitute means ± S.D. (*n* = 6). Spermatozoa extracts from the control, Cr 0.5, and Cr 1 groups were immunoblotted for Hsp90, Hsp70, Hsp60, and β-actin. Representative immunoblots are shown. The asterisk (*) denotes statistically significant differences compared to the control, while the caret (^) denotes statistically significant differences between the crocin groups (*p* < 0.05).

The effect of crocin on different Hsp levels in bovine spermatozoa is presented in Figure 3. The results indicate that crocin 0.5 mM did not affect Hsp90 levels after freeze/thaw process, while the Cr 1 group significantly (*p* < 0.05) upregulated the expression of Hsp90, compared to both the control and the Cr 0.5 group (Figure 3A). On the contrary, Hsp60 and Hsp70 levels were increased dose-dependently in the crocin-treated groups (*p* < 0.05) compared to the control. Statistically significant (*p* < 0.05) differences were also observed among the crocin groups (Figure 3B,C).

### 2.4. Cryopreservaton in the Presence of Crocin Protected Bovine Spermatozoa by Inhibiting the Induction of Apoptosis

**Figure 4:**
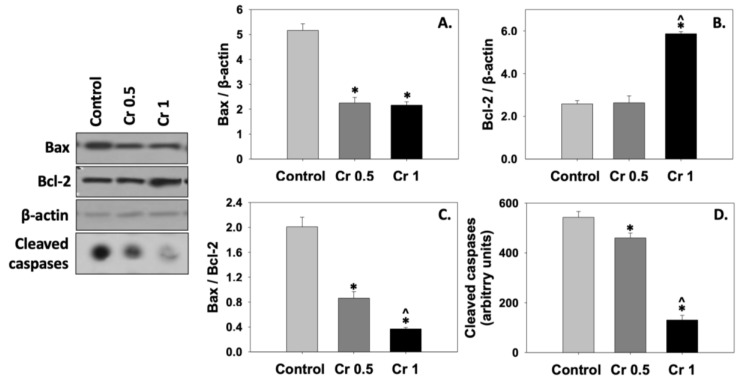
Bax (**A**), Bcl-2 (**B**), Bax/Bcl-2 ratio (**C**), and cleaved caspase substrate (**D**) levels in bovine spermatozoa under the effect of 0.5 mM (Cr 0.5) or 1 mM (Cr 1) crocin. Values constitute means ± S.D. (*n* = 6). Spermatozoa extracts from the control, Cr 0.5, and Cr 1 groups were immunoblotted for Bax, Bcl-2, cleaved caspase substrates, and β-actin. Representative immunoblots are shown. The asterisk (*) denotes statistically significant differences compared to the control, while the caret (^) denotes statistically significant differences between the two crocin groups (*p* < 0.05).

Bax and Bcl-2 protein levels and the Bax/Bcl-2 ratio were determined as apoptosis-related biomarkers. The levels of Bax protein were significantly (*p* < 0.05) decreased in spermatozoa frozen in the presence of either crocin concentration (0.5 and 1 mM) compared to the control group (Figure 4A). On the other hand, Bcl-2 levels remained unchanged in the Cr 0.5 group, while supplementation with 1 mM crocin significantly (*p* < 0.05) increased Bcl-2 levels compared to the control and Cr 0.5 groups (Figure 4B). Therefore, the Bax/Bcl-2 ratio (Figure 4C) was significantly (*p* < 0.05) decreased in both Cr 0.5 and Cr 1 groups compared to the control. This anti-apoptotic effect of crocin was further verified by a decrease in the levels of cleaved caspase substrates (Figure 4D). Statistically significant differences were also observed between the two crocin groups (Cr 0.5 and Cr 1), thus indicating a dose-dependent anti-apoptotic effect of crocin (Figure 4B–D).

## 3. Discussion

In an effort to understand the physiological response of spermatozoa to stress and oxidative stress in particular, we studied their response to the cold stress they sustain during the freeze/thaw process. Previous studies by our group have demonstrated that the addition of crocin to the freezing medium improves sperm characteristics, and this appears to be mediated via the mitigation of the oxidative stress induced during freezing [26]. While the existing literature suggests that crocin supplementation either during freezing or during thawing ameliorates sperm quality parameters after freeze/thaw [19,26,27,28,29,30,31,32], no attempt was made to approach the mechanism(s) by which crocin exerts its protective role. Therefore, taking into consideration the fact that antioxidants, including crocin, may act in different ways [36], the aim of this study was to elucidate the cryoprotective biochemical role of crocin as an antioxidant compound of the freezing medium of spermatozoa and investigate the molecular events that are involved in antioxidant capacity, apoptosis, and the HSR.

### 3.1. Free Radical Scavenging Activity of Crocin

A controlled production and removal of O_2_^−•^ (to ensure optimal levels) are necessary to attain crucial reproductive processes, especially in spermatozoa [6]. However, this free radical is also involved in the initiation of LPO. In the present study, the addition of 1 mM crocin in the freezing medium suppressed the levels of superoxide anion after the freeze/thaw process. This result complies with the decreased LPO observed in these spermatozoa, following their incubation at 37 °C [26,31], as well as with other studies in cryopreserved goat spermatozoa, where 1 mM crocin decreased the levels of O_2_^−•^ [29], or in buffalo spermatozoa, where a dose-dependent effect of crocin in scavenging O_2_^−•^ has been suggested [30]. In the latter study, four concentrations of crocin (0, 0.5, 1, and 2 mM) were employed, and the data suggest that all concentrations were effective, with the highest concentration of crocin (2 mM) being the most effective [30].

Additionally, spermatozoa that were frozen in the presence of crocin exhibited higher TAC. Similar observations have been reported in different cell types and under diverse conditions, such as in liver cells of diabetic rats [22] and hepatocellular carcinoma cells [37]. More importantly, a positive effect of crocin on TAC has also been demonstrated in human [32] and rooster [31] spermatozoa. Salehi and co-authors suggested that crocin is a robust compound which enhances TAC in human spermatozoa, while the addition of 1 mM crocin in the extender of rooster spermatozoa resulted in higher levels of TAC and decreased LPO [32]. Spermatozoa are highly vulnerable to oxidative stress, as they rely on high mitochondrial function to maintain their mobility. Thus, high amounts of ROS are produced, culminating, under stress conditions, in the accumulation of oxidative byproducts which damage the cell membranes, proteins, and DNA [8,9]. The radical scavenging activity of crocin, and its potential to enhance the antioxidant capacity of spermatozoa, explain why crocin suppresses LPO and maintains optimum levels of motility and viability after the freeze/thaw process [27,31,32].

### 3.2. Crocin Inhibits Free Radical-Generating Enzymes and Activates Intracellular Antioxidant Enzymes

The induction of iNOS in spermatozoa is responsible for increased NO production and alterations in sperm physiology caused by nitrosative stress. The latter is implicated in mitochondrial dysfunction, DNA fragmentation, and apoptosis [38]. Despite the significance of this observation, to the best of our knowledge, this is the first report on the effect of crocin on the activity and/or protein levels of iNOS in spermatozoa. In the present study, it was demonstrated that crocin decreased the protein levels of iNOS to levels similar to those of control, thus further establishing an antioxidant role of crocin in this system. This is possibly attributed to the inherent antioxidant properties of crocin, as another potent antioxidant, melatonin, exerts a similar effect on bovine spermatozoa [39]. Crocin also modulates iNOS expression in several physiopathological systems with a redox imbalance, such as lipopolysaccharide-(LPS)-challenged murine macrophages [40], rat post-ischemic cardiac cells, and myocardial infarction [41].

The activation of intracellular antioxidant enzymes in spermatozoa decreases the consequences of ROS attack and oxidative stress [8]. The present results have shown that the addition of crocin stimulates the expression of SOD-1, thus reducing the levels of O_2_^−•^. Of course, this could result in an accumulation of H_2_O_2_, as SOD-1 reduces O_2_^−•^ into H_2_O_2_ and O_2_, which in turn could be harmful for spermatozoa. However, this carotenoid also affects the levels of CAT and/or GPx, thus eliminating H_2_O_2_ before the generation of ^▪^OH through the Fenton reaction [23,42]. This hypothesis is supported by recent findings in cryopreserved human spermatozoa in the presence of 1mM crocin, where the accumulation of H_2_O_2,_ was deteriorated and glutathione content increased [32]. A possible effect of crocin on the expression of CAT and GPx in bovine spermatozoa would be worth investigating. The results of the present study could partially explain this effect, since the GSH content of bovine spermatozoa has been increased due to the addition of crocin. This effect has also been verified for other antioxidant compounds, such as melatonin and pterostilbene, that enhance GSH synthesis or limit its depletion [39,43]. The impact of crocin on glutathione-related processes is very important since GSH can act directly as an antioxidant to protect sperm cells against free radicals, such as ONOO^−^, singlet oxygen (^1^O^2^), and H_2_O_2_ [44], and as a cofactor for antioxidant and detoxification enzymes, such as GPx [44,45].

### 3.3. Crocin Modulates the Apoptotic Cascade and HSR

Oxidative stress stimulates the formation of lipid hydroperoxides, including malondialdehyde, which is the most toxic, with subsequent deleterious effects on the fertilizing capacity of spermatozoa [8]. LPO disrupts the integrity of cell membranes and induces DNA fragmentation and apoptosis. The protective effect of crocin, when added in the extender, was reflected by a lower percentage of (early) apoptotic spermatozoa [29,31] and decreased LPO levels [27,31]. In Yanbian yellow cattle spermatozoa, the addition of 1 mM crocin to the freezing medium resulted in increased levels of the anti-apoptotic Bcl-2 and decreased levels of pro-apoptotic Bax. Consequently, the Bcl-2/Bax ratio increased, showing that crocin is a cryoprotectant factor that decreases cell apoptosis during freeze/thaw process [19]. The mRNA expression of Bcl-2 was also increased in rooster spermatozoa cryopreserved in the presence of 1 mM crocin, accompanied by a decrease in caspase-3 levels [31]. The results of the present study are in line with these observations since the addition of 1 mM crocin resulted in increased Bcl-2 levels, while both concentrations significantly decreased Bax levels. Crocin seems to dose-dependently affect the susceptibility of spermatozoa to apoptosis by affecting the Bax/Bcl-2 ratio. In the same context, it inhibits activation of caspases, the main units of apoptotic machinery, documented by the decrease in cleaved caspase substrates levels. The beneficial anti-apoptotic role of crocin is also exhibited in other systems, such as myocardial oxidative stress induced by streptozotocin in *Rattus norvegicus* or cardiomyocyte oxidative stress induced by high glucose [17].

The levels of Hsps after cryopreservation are substantially decreased due to an increase in ROS and eventual oxidative damage of intracellular enzymes [18]. In the present study, both concentrations of crocin (0.5 mM and 1 mM) upregulated Hsp60 and Hsp70 levels. The expression of Hsp60 is induced in cells exposed to heat/cold stress to mediate a cryoprotective role, mainly through the regulation of apoptosis [16,18]. The literature regarding the role of Hsp60 in sperm physiology is very limited. However, in cancer cells, Hsp60 mediates an anti-apoptotic role by interacting with Bax [46]. Therefore, as anticipated, in the present study, both Bax levels and apoptosis were suppressed, while Hsp60 levels were dose-dependently upregulated by crocin. Moreover, the abundance of Hsp70 is closely related to the fertilizing capacity in water buffalo spermatozoa [47]. This protein is located in the membrane of the midpiece and the mitochondria of bovine sperm. There, it functions as a cell protector of membrane fluidity and energy regulator for sperm motility. This implies that decreased Hsp70 levels are associated with membrane rigidity and decreased motility [35,48]. Moreover, a positive correlation between Hsp70 and SOD-1 exists (also depicted for crocin 1 mM in the present study) and implies that this protein is also involved in promoting survival against oxidative stress-dependent damage [18]. In a nutshell, the upregulation of Hsp70 can help spermatozoa become more resistant to the damaging effects of cryopreservation, preserving sperm motility and post-thaw fertility, as has been shown in buffalo spermatozoa.

The levels of Hsp90, the most abundant protein in the cytoplasm of eukaryotic cells [49], were also positively affected by crocin. Hsp90 content is correlated with sperm motility and viability, as well as plasma membrane and acrosome integrity, in boar and bull sperm [50,51,52]. Moreover, Hsp90 has been shown to repair chromosomal damage caused by the freeze/thaw process and maintain DNA integrity in boar spermatozoa [53]. The increased levels of Hsp90 by crocin can be associated with the preservation of the motility and viability of spermatozoa that were cryopreserved in the presence of 1 mM crocin [27]. Similar observations have been recorded for melatonin, another potent antioxidant, in cryopreserved human semen [54].

The relationship between crocin and Hsps seems quite intriguing. While it would be expected for crocin to decrease Hsp levels, since it modulates oxidative stress and enhances the antioxidant capacity of spermatozoa, the addition of crocin upregulates the Hsp members examined herein. We suggest that crocin may increase the expression of the aforementioned Hsps and both factors, crocin and Hsps, may act synergistically to facilitate the adaptation of cells against stress factors and counteract the consequences of cryopreservation. The addition of crocin ensures controlled amounts of ROS, which implies that Hsps can focus on the maintenance of homeostasis and cell viability.

Although the data presented clearly demonstrate the protective antioxidant effect of crocin on cryopreserved spermatozoa, the main limitation of the experimental set up is the relatively small number of bull sperm donors used in this study and hosted in the Department of Reproduction and Artificial Insemination in Ionia. This limits the number of genetically different semen samples used in this study. Another limitation is the difficulty we encountered in quantifying the ROS and RNS, probably because they are highly reactive and unstable, thus yielding inconclusive results. Finally, it has not been possible to provide a conclusive description of the signaling pathway implicated due to the complexities of related and interacting pathways implicated in cell death or survival, such as those mediating apoptosis, ferroptosis, autophagy, and mitophagy. Further studies are required to describe the implicated processes in a detailed, documented manner.

## 4. Materials and Methods

### 4.1. Sperm Collection and Freezing Protocol

Sperm samples were collected in spring 2019 from 4 healthy and sexually mature bulls (2 Simmental, 1 Holstein, 1 Limousin) of proven fertility, trained to a semen collection routine, as previously described [27]. Experimental procedures and animal care conditions followed the national and European Union directive recommendations (86/609/EEC). No additional handling of sperm donors was performed for this experiment; thus, no approval from an ethical committee was necessary.

The samples were collected and evaluated before cryopreservation to meet standard criteria (>70% total motility, >75% viability, and a total concentration of at least 4 × 10^9^ spermatozoa × mL^−1^). The semen from each ejaculate was divided into three parts and mixed with a home-made Tris-egg yolk extender (20% Tris-egg yolk, 7% glycerol, 78 mM of citric acid, 69 mM of fructose, 50 μg of tylosin, 250 μg of gentamycin, 150 μg of lincomycin, 300 μg of spectinomycin mL^−1^) to a final concentration of 50 × 10^6^ spermatozoa × mL^−1^ according to a previously described method [27]. For the purpose of this study, crocin was added to the two parts to a final concentration of 0.5 mM (Cr 0.5) or 1 mM (Cr 1), while the third part served as a control. Subsequently, the straws were cooled to 5 °C for 4 h, and then they were transferred into a freezing chamber (Digital cool alpha, IMV Technologies, Shanghai, China), where the temperature had been set to −12 °C. The straws were then placed on a horizontal rack, 3 cm above the surface of liquid nitrogen, to reach −100 °C within 10 min. Finally, the straws were plunged into liquid nitrogen (−196 °C) for 5 min and transferred to sperm tanks.

For each experiment, the appropriate number of straws from each of the 4 animals were thawed via immersion in a water bath (37 °C for 40 s) and pooled. The pool of thawed spermatozoa was layered onto a discontinuous Percoll gradient (45% and 80%) and centrifuged at 380× *g* for 25 min at room temperature (RT) to remove the cryoprotectants. The supernatant was carefully removed, and the pellet was resuspended in Sperm Tyrode’s Albumin Lactate Pyruvate (TALP) solution (100 mM NaCl, 3.1 mM KCl, 25 mM NaHCO_3_, 0.29 mM NaH_2_PO_4_, 21.6 mM sodium lactate, 2 mM CaCl_2_, 1.5 mM MgCl_2_, and 10 mM HEPES sodium salt, supplemented with 1 mM sodium pyruvate and 50 μg mL^−1^ gentamycin) and centrifuged at 300× *g* for 10 min (RT). Six thawing cycles were performed with samples from all 4 bulls, and all assays were repeated 6 times (*n* = 6).

### 4.2. Determination of Total Antioxidant Capacity

The total antioxidant capacity (TAC) of spermatozoa was determined by a 2,2-Diphenyl-1-picrylhydrazyl radical (DPPH^•^) scavenging assay as previously described [55]. Spermatozoa (5 × 10^6^) in Phosphate Buffer (10 mM KH_2_PO_4_,10 mM Na_2_HPO_4_, pH 7.4) were subjected to two cycles of sonication at 28 kHz for 60 s to prepare the cell lysates. Subsequently, DPPH^•^ (0.08 mM) was added, and the samples were incubated at RT, in the dark, for 60 min. The absorbance of the blank (Phosphate Buffer with 0.08 mM DPPH^•^ without cell lysates) and the residual DPPH^•^ in the samples were measured at 517 nm using a spectrophotometer (Pharmacia LKB-Novaspec II, Northwich, Cheshire, UK). The antioxidant capacity of spermatozoa (%) in each sample was determined as follows: [(Absorbance_Blank_ − Absorbance_sample_) / Absorbance_Blank_] × 100.

### 4.3. Determination of Intracellular Glutathione (GSH)

Intracellular reduced glutathione (GSH) levels were determined as previously described [39]. Lysates of spermatozoa (20 × 10^6^) in Phosphate Buffer (67 mM KH_2_PO_4_, 67 mM Na_2_HPO_4_, pH 8) were prepared as described above (see Determination of TAC). The lysates were incubated with 0.33 mM DTNB [5,5′-dithiobis (2-nitrobenzoic acid)] for 60 min at RT, in the dark. The absorbance was measured at 412 nm with a spectrophotometer (Pharmacia LKB-Novaspec II, Northwich, Cheshire, UK).

### 4.4. Quantification of the Superoxide Anion (O_2_^−^) Production

Spermatozoa (2 × 10^6^) were incubated with 6 μM of NBT [2,20-bis(4-Nitrophenyl)-5,50-diphenyl-3,30-(3,30-dimethoxy-4,40 diphenyleneditetrazolium chloride)] for 60 min at 37 °C in the dark [55]. Ending incubation, the residual NBT solution was removed by centrifugation, leaving a pellet containing the cells and blue formazan crystals, which were dissolved in 2 M KOH (120 μL) and dimethyl sulfoxide (DMSO) (140 μL). A 96-well microplate (BioTek EL800, Thomas Scientific, Swedesboro, NJ, USA) photometer was used to measure optical density at 630 nm. Data are presented as percentage of the control sample, which was set to 100%.

### 4.5. SDS-PAGE/Immunoblot and Dot Blot Analysis

Percoll gradient-isolated spermatozoa were prepared for immunoblotting and dot blot analysis for the detection of Hsp90, Hsp70, Hsp60, Bax, Bcl-2, cleaved caspases, iNOS, SOD-1, and β-actin according to well-established protocols, as previously described in detail [39,55]. The antibodies employed in the present study were the following: anti-heat shock protein, 70 kDa (Cat. No. H5147, Sigma, Darmstadt, Germany), anti-heat shock protein, 90 kDa (Cat. No. H1775, Sigma, Darmstadt, Germany), anti-heat shock protein, 60 kDa (Cat. No. 12165, Cell Signaling, Beverly, MA, USA), anti-Bcl2 (2872, Cell Signaling, Beverly, MA, USA), anti-Bax (B-9) (2772, Cell Signaling, Beverly, MA, USA), anti-iNOS (#18985-1-AP, Proteintech, Manchester, UK), anti-SOD1 (#10269-1-AP, Proteintech, Manchester, UK), and anti-cleaved caspase substrate motif (8698 Cell Signaling, Beverly, MA, USA). Actin (anti-β actin 3700, Cell Signaling, Beverly, MA, USA) was employed for quality transfer control and normalization.

### 4.6. Statistical Analysis

One-way analysis of variance (ANOVA) (GraphPad Prism 8, GraphPad Software Inc., GraphPad Software Inc., La Jolla, CA, USA) followed by Bonferroni post hoc were employed to test for significance at *p* < 0.05 (5%) level between the three experimental groups examined in the present study: control, Cr 0.5, and Cr 1. Bars represent mean values ± standard deviation (S.D.) from six independent experiments.

## 5. Conclusions

The addition of crocin in the freezing medium protects spermatozoa against cold stress and the disturbances it causes to sperm structure and function. Crocin mediates these effects by enforcing the antioxidant capacity of bovine spermatozoa and inhibiting the apoptotic pathways related to the freeze/thaw process. This ameliorative effect can be attributed to the activation of antioxidant enzymes, an increase in the ROS scavenging activities, the inhibition of iNOS, the induction of the HSR mechanisms of spermatozoa, and the induction of anti-apoptotic proteins.

## Data Availability

Data are contained within the article.

## Abbreviations

The following abbreviations are used in this manuscript:

iNOS	inducible nitric oxide synthase
CAT	catalase
GPx	glutathione peroxidase
SOD-1	superoxide dismutase-1
Hsp	heat shock proteins
HSR	heat shock response
LPO	lipid peroxidation
ROS	reactive oxygen species
RT	room temperature
TAC	total antioxidant capacity
GSH	reduced glutathione
